# Understanding lung cancer screening motivation: cognitive, affective, and thought-based predictors in a UK screening-eligible sample

**DOI:** 10.1093/abm/kaag031

**Published:** 2026-06-19

**Authors:** Andriana Theodoropoulou, Olayinka Farris, Isaac Chung, Sheina Orbell

**Affiliations:** Department of Psychology, University of Essex, Colchester CO4 3SQ, United Kingdom; Essex ESNEFT Psychological Research Unit for Behaviour, Health and Wellbeing, University of Essex, Colchester CO4 3SQ, United Kingdom; Department of Psychology, University of Essex, Colchester CO4 3SQ, United Kingdom; Essex ESNEFT Psychological Research Unit for Behaviour, Health and Wellbeing, University of Essex, Colchester CO4 3SQ, United Kingdom; Department of Psychology, University of Essex, Colchester CO4 3SQ, United Kingdom; Essex ESNEFT Psychological Research Unit for Behaviour, Health and Wellbeing, University of Essex, Colchester CO4 3SQ, United Kingdom; Department of Psychology, University of Essex, Colchester CO4 3SQ, United Kingdom; Essex ESNEFT Psychological Research Unit for Behaviour, Health and Wellbeing, University of Essex, Colchester CO4 3SQ, United Kingdom

**Keywords:** cancer screening, health behavior, theory of planned behavior, attitude, regret, moral norm

## Abstract

**Background:**

A nationwide targeted lung cancer screening program is underway in the United Kingdom, but uptake in recent trials remains modest, and little is known about the psychological processes that might influence uptake.

**Purpose:**

This study aimed to understand how individuals respond to a lung cancer screening invitation by identifying the key psychological, cognitive, and affective variables shaping screening intention and the thought processes underlying them.

**Methods:**

UK screening-eligible current and former smokers aged 55-74 years (*N *= 1106) read an online lung screening invitation before providing their unprompted thoughts and completing measures of cognitive and affective variables (attitude; descriptive, subjective, and moral norms; perceived behavioral control; fear; anticipated regret; and fatalism). Logistic regressions examined predictors of screening intention, with follow-up analyses linking thought content to significant cognitive and affective predictors.

**Results:**

Moral and descriptive norms, perceived behavioral control, and inaction regret were positively associated with intention, while fear and anticipated regret associated with taking part and receiving abnormal results were negatively associated. Thoughts reflecting NHS program doubts, stigma, and cancer worry were consistently negatively associated with cognitive and affective predictors, whereas perceived screening benefits of early detection and peace of mind, and personal/family cancer history were positively associated.

**Conclusion:**

Screening motivation was shaped by both cognitive and affective factors, underpinned by distinct thoughts. Findings suggest that health communication interventions may strengthen moral and descriptive norms, leverage inaction regret, and enhance self-efficacy by addressing feelings of stigma, embarrassment, and cancer worry to increase screening participation at this critical stage of national implementation.

Lung cancer is the leading cause of cancer death worldwide, with over 4000 people diagnosed annually in the United Kingdom.^1^^,^^2^ Since symptoms often emerge only at advanced stages, prognosis is typically poor, with 5-year survival rates of only 12%-16%.^3^ Crucially, early-stage detection significantly improves survival rates to 60%-70%,^4^ with large randomized controlled trials demonstrating that targeted screening through low-dose computed tomography (LDCT) reduces lung cancer mortality by 20%-24%.^1^^,^^5^^,^^6^ In the United Kingdom, the National Health Service (NHS) Lung Cancer Screening (LCS) Program, known in its earlier pilot phase as the Targeted Lung Health Check (TLHC) Program, is currently underway in selected areas and is expected to expand nationwide by 2029.^2^^,^^7^ Unlike UK screening programs for bowel, breast, and cervical cancer, the LCS program targets individuals based on a behavioral characteristic. The program invites higher-risk adults—current and former smokers aged 55-74 years—to an initial Lung Health Check (LHC), with LDCT offered to those deemed at an elevated risk of developing lung cancer based on further risk stratification using multivariable model risk threshold (Liverpool Lung Projectv2 (LLPv2) or Prostate Lung Colorectal Ovarym2012 (PLCOm2012) models. Uptake in recent pilot trials has been modest, ranging between 25% and 50%,^8^^,^^9^ while uptake in the initial program rollout is 49%.^10^ Understanding how people respond to an initial screening invitation and the psychological processes that shape their decision to accept the invitation to a telephone health check with a health professional is therefore crucial for invitation and message design to increase screening participation as the NHS Lung Cancer Screening Program moves toward national implementation.

## Overview and limitations of existing psychological research

Existing psychological research, largely from qualitative and retrospective studies, has suggested a range of barriers and facilitators that might influence engagement with lung cancer screening. Barriers include worry about cancer diagnosis and cancer-related anxiety,^11–14^ fatalistic beliefs,^13–18^ smoking-related stigma,^14^^,^^15^^,^^17^^,^^19^ low perceived efficacy of screening,^15^ low perceived cancer risk,^11^^,^^14^^,^^17^ concerns about the screening process itself,^11^^,^^14^^,^^16^ mistrust of healthcare professionals or systems,^11^^,^^15^^,^^19^^,^^20^ and practical barriers, such as cost, access, and time constraints.^11^^,^^12^^,^^14^^,^^19^ By contrast, facilitators include perceived benefits of early detection^14^^,^^17^^,^^19^^,^^21^ and peace of mind,^14^^,^^15^^,^^19^ high perceived cancer risk,^11^ seeing screening as an opportunity to support smoking cessation,^14^^,^^17^^,^^19^ and healthcare provider recommendation.^12^^,^^15^^,^^20^ However, much of prior research has usually been constrained by several methodological limitations. Many studies have relied on small, qualitative designs,^12^^,^^14^^,^^16^^,^^17^^,^^19^ retrospective accounts of non-attenders^11^ or those already screened,^17^ samples focusing on clinicians’ perspectives,^13^ and non-UK samples.^16^^,^^19–21^ Although these approaches provide valuable insights, they are limited in their ability to quantify the relative contribution of different psychological variables on lung screening intention, or to generalize findings to the wider screening-eligible population in the United Kingdom. The present study addresses these limitations by employing a large, screening-eligible, prospective UK sample and a comprehensive set of psychological measures to identify the key determinants of lung cancer screening motivation and intention.

## Cognitive and affective psychological determinants

Psychological cognitive constructs from the Theory of Planned Behavior (TPB)^22^ have been consistently linked to cancer screening decisions. In cervical, colorectal, and breast cancer screening contexts, more positive attitudes toward screening, stronger perceived social norms, and greater perceived behavioral control (PBC) have been associated with stronger intentions to attend screening and, in some cases, actual screening participation.^23–28^ TPB posits that intentions are shaped by attitude, subjective norms, and perceived behavioral control (PBC). Research applying a full TPB model in lung cancer screening, however, remains limited to one small US-based study focusing on ethnic differences,^29^ while associations between TPB constructs and lung cancer screening intention have mostly been explored indirectly through qualitative studies targeting specific constructs (eg, attitudes).^13^^,^^17^^,^^18^ The present study aims to address this gap by assessing attitudes, subjective norms, and perceived behavioral control to determine their predictive power in lung cancer screening intention.

In addition to subjective norms, research suggests that descriptive norms (what similar others do) can independently influence both screening intention and actual uptake in colorectal cancer screening and is amenable to intervention,^30^^,^^31^ but research on lung cancer screening remains limited. Family norms–perceptions of close relatives’ expectations and support–have been implicated as both a facilitator and barrier to lung cancer screening, but findings have so far been constrained by small, qualitative samples targeting clinicians’ perspectives.^13^ General Practitioner (GP) norms have also emerged as a determinant: a qualitative study of screening-eligible adults found that physician recommendation was a key influence on decisions to screen for lung cancer,^12^ while a quantitative US study testing a conceptual model found that clinician recommendation was directly associated with screening participation.^20^ A further extension is moral norm, a sense of personal obligation, which adds unique explanatory power to intention across health behaviors,^32^^,^^33^ and which remains unexplored in the context of lung cancer screening. To the best of our knowledge, no study has yet simultaneously tested the effects of different normative components on lung cancer screening intention. Accordingly, the present study includes different types of norms to identify which, if any, best predict screening intention.

Affective variables also contribute to screening decisions. Anticipated regret, defined as the anticipated negative affective consequences of “not acting,”^34^ has been found to predict cancer screening participation decisions in both colorectal^35^ and cervical^36^ cancer. Anticipated negative affect in the context of screening has also been proposed to occur in relation to “acting” and to obtaining either an abnormal or a normal result.^34^ However, the influence of anticipated regret in lung cancer screening remains limited, so we assessed each of these forms of anticipated regret. Affect associated with the thought of developing disease is also an important component of accounts of health-related behavior, such as protection motivation theory.^37^ Fear of cancer, in particular, has been identified as both a motivator^21^ and a deterrent^11^^,^^14^ to lung cancer screening intentions, with one study reporting no association at all,^15^ but findings have so far been constrained by qualitative^14^ or retrospective designs^11^ and non-UK samples,^21^ thus necessitating the need to further unpack these mixed effects in a UK sample. Fatalism, the belief that outcomes are inevitable or beyond personal control, has also emerged as a barrier to lung cancer screening participation in qualitative studies.^14^^,^^18^ Taking everything together, we included a comprehensive list of cognitive and affective predictors, some of which have never been tested before in the context of lung cancer, to identify the key psychological determinants of lung cancer screening intention.

## Thought listings

One unexplored area to further tap into the psychological determinants of screening intention is through individuals’ spontaneous thoughts when reading a lung cancer screening invitation and how these relate to significant cognitive and affective predictors of intention. The cognitive response approach^38^^,^^39^ proposes that when presented with a persuasive communication, in this instance, an invitation providing information about a new lung health check, people will access their own prior beliefs and understanding, scrutinize message content, make inferences, and generate new arguments about that communication.^40^ They may accept message content such as possible benefits of screening, consider its self-relevance, and produce counter-arguments to the message content. In the present study, we aimed to capture the content of these thoughts generated after reading an invitation. A thought-listing approach allows us to capture these salient beliefs at the point of the screening invitation processing, thereby offering novel insight into which thoughts underlie the key cognitive and affective predictors of intention and how these variables might be changed. For instance, communication interventions can be tailored to foster thoughts associated with positive predictors of intention and reduce thoughts associated with negative predictors. For this reason, we examined not only which cognitive and affective variables shape screening intention, but also the unprompted thoughts that statistically underlie these variables.

## The present study

The present study employs a large UK sample of screening-eligible adults to examine spontaneously generated thoughts to a lung-screening invitation alongside measures of cognitive and affective variables. Specifically, we had 2 main aims. First, we wanted to examine how cognitive and affective variables shape screening intention. Second, we wanted to identify the thoughts that underlie the significant cognitive and affective psychological predictors. By integrating theory-driven constructs with spontaneous cognitions, this enables us to provide evidence-based recommendations for how screening invitations might be refined to target significant predictors of intention and foster the kinds of thoughts most likely to support lung cancer screening participation at this critical stage of national implementation.

## Methods

### Ethics

The study received ethical approval from the Ethics Committee at the University of Essex (ref: ETH2425-0226) and complied with all relevant ethical regulations. Informed consent was obtained from all participants, and participants were reimbursed £1.50 on Prolific for their participation.

### Participants

Participants (*N *= 1106) were recruited online via the research platform Prolific between January 2025 and June 2025. Eligibility was based on NHS criteria for lung health checks: current and former smokers; aged between 55 and 74; and registered with a GP in England. An initial 1188 responses were collected. Participants who had a completion score below 90% or had failed both attention check questions were subsequently removed, resulting in a final analytical sample of *N *= 1106.

### Study design and procedure

This was a cross-sectional, mixed-methods, online study delivered via Qualtrics. Participants were first presented with the lung cancer screening invitation and were asked to list the thoughts that came to their mind after reading the text. They then completed several measures of cognitive and affective psychological variables (attitude; perceived behavioral control; and subjective, descriptive, and moral norms; anticipated regret; fear; and fatalism), with lung screening intention being the main dependent variable (see Materials S1 for full details). All analyses were conducted using SPSS version 29. Hierarchical logistic regression was performed to identify the predictors of screening intention by entering socio-demographic variables in Step 1, smoking status in Step 2, and cognitive and affective variables in Step 3. Follow-up multiple linear regressions were conducted to identify which thought types predicted the cognitive and affective significant predictors.

### Materials

#### Demographics

Participants completed several socio-demographic questions (eg, education, age, income, wealth, occupation, ethnicity, Wardle items, and IMD), and some health-related questions (eg, smoking history and comorbidities).

#### Lung screening invitation

The screening invitation materials were developed by the research team and informed by the NHS website and existing NHS communication materials, such as the lung health check leaflet. Participants were first presented with information regarding the prevalence and nature of lung cancer and the introduction of the lung health check. Following this, they were presented with a balanced mix of possible benefits (peace of mind, early detection, and motivation for healthier lifestyle changes) and costs (worry about cancer, potential effect on close others, and potential follow-up unpleasant procedures) associated with taking part in lung cancer screening (see Materials S1 for the full invitation materials).

#### Thought listings

Immediately after reading the screening invitation, participants completed a thought-listing task. They were prompted with the question: “*Please write down the thoughts that come to mind as you read the paragraphs above*.” The thoughts listed by participants were first independently coded by the first and second author as either positive (favorable evaluation toward the lung health check) or negative (unfavorable evaluation). Interrater reliability was excellent for both positive (*k* = .93) and negative (*k* = .92) thoughts. To identify specific thought content, an inductive thematic approach was used. All responses were initially reviewed by the first and second authors to generate preliminary codes and identify recurring patterns in the data. Through an iterative process of coding and comparison, these initial codes were grouped into higher-order categories, resulting in a coding framework comprising 15 distinct thought types. All responses were then systematically recoded by the first author using this framework, with each thought type coded as present (1) or absent (0) for each participant to allow for quantitative content analysis. The second author reviewed a subset of responses to ensure consistency and reliability in the application of the coding frame, with any discrepancies addressed and resolved through discussion. The full coding framework is provided in Materials S2.

#### Cognitive and affective measures

After completing the thought listings, participants responded to items assessing cognitive and affective constructs. Direct evaluative measures^41^ from the cognitive constructs of the theory of planned behavior,^22^ including attitude, subjective norm, perceived behavioral control, and intention, were operationalized according to standard procedures and adapted from prior screening research.^24^^,^^26^^,^^34^^,^^42^  *Attitude* toward participating in the LHC was assessed using the stem: “For me, to take part in a lung health check if it were offered to me in the next 12 months would be…” Participants responded on 12 bipolar adjective scales: worthless–worthwhile, unnecessary–necessary, bad–good, unimportant–important, unpleasant–pleasant, harmful–beneficial, ineffective–effective, intrusive–non-intrusive, embarrassing–comfortable, annoying–pleasing, foolish–wise, and useless–useful. The overall attitude scale demonstrated high internal consistency (α = .93). *Subjective norms* were measured using 4 items across 3 different subscales tapping into referents (what most people who are important to me would think), GP norms (what my GP would think), and family norms (what my family would think). An example item was: “If I were offered a lung health check in the next 12 months, most people important to me would think I should take part.” Internal consistency was high for all: subjective norms (α = .93), GP norms (α = .94) and family norms (α = .94). *Descriptive norm* was assessed using a single item: “Thinking about people of your age and gender that you know, how many of them do you think will take part in a lung health check if it is offered to them within the next 12 months?” (1 = none of them to 5 = almost all of them). *Moral norm* was assessed using 4 items (eg, “I have a moral obligation to take part in a lung health check if it is offered to me,” “It would be morally right for me to take part in a lung health check if it is offered to me,” “I would feel guilty if I receive an invitation for a lung health check and did not go,” “I feel I ought to take part in a lung health check if I get offered it”). Internal consistency was α = .89. *Perceived behavioral control* was assessed using 7 items. An example item was: “If I were offered a lung health check in the next 12 months, I am confident/unconfident that I could take part …” (confident–unconfident). The scale showed internal consistency of α = .87. Affective constructs tapping anticipated regret and fear were measured using validated scales adapted from prior screening research.^34^^,^^36^  *Anticipated regret* for screening was measured using 4 items for each of 3 scenarios: not taking part (eg, “If I am offered the chance to take part in the lung health check in the next 12 months and I do not take part, I will feel…”), taking part and receiving normal results (eg, “… If I take part and no problems are found, I will feel…”), and taking part and receiving abnormal results (eg, “… if I take part and lung cancer is found and treated, I will feel…”). Affective responses were rated using scales such as regretful–not regretful, relieved–not relieved, sorry–not sorry, worried–not worried, and ashamed–not ashamed. Internal consistency was α = .89 for nonattendance, α = .83 for normal results, and α = .82 for abnormal results. *Fear* of lung cancer was assessed with the stem: “The thought of getting lung cancer makes me feel….” Responses included very scared–not at all scared, very anxious–not at all anxious, very frightened–not at all frightened, and very ashamed–not at all ashamed. Internal consistency was α = .84. All scale scores were averaged, yielding values between 1 and 6 for each construct. Items were coded such that higher scores reflected greater positive motivation and attitudes, as well as greater negative affect. Finally, cancer fatalism was measured using the Powe Cancer Fatalism Scale.^43^ An example item was: “I think if someone has cancer, it is already too late to get treated for it.” Internal consistency was α = .92.

#### Intention

Screening intention was measured with 6 items (eg, “I intend to take part in a lung health check if it were offered to me in the next 12 months”) via a (1 = Strongly disagree, 6 = Strongly agree) scale, with higher scores reflecting higher intention. Internal consistency was α = .99. Due to an overall high sample intention score (*M *= 5.27), intention was subsequently coded into a binary (intenders vs non-intenders) variable for our analyses.

## Results

The full participant characteristics of our analytical sample (*N *= 1106) are presented in Table 1. The sample age ranged from 54 to 74 years *(M *= 61.7, *SD *= 5.1), with 53.8% women, 46.1% men, and 0.1% self-described gender, while most identified as White (96.3%), and 41.0% held university degrees. Based on self-reported postcodes, an Index of Multiple Deprivation (IMD) decile was assigned to each participant. Of the 1092 participants with valid postcode data, 49.3% were classified within the most deprived 5 deciles (1-5), and 50.7% within the least deprived 5 deciles (6-10). Most participants (64.4%) indicated *yes* to all 3 Wardle et al^44^ items (house and car ownership, and any educational qualification). A smoking status variable was created for our analyses: 24.1% were classified as recent smokers (having smoked within the past month), 21.3% as short-term past smokers (having smoked between 1 month and 10 years ago), and 54.6% as long-term past smokers (having smoked more than 10 years ago). For the logistic regression analyses, some socio-demographic variables were subsequently coded into binary predictors: gender (male vs female), living situation (living alone vs with others), education (below degree level vs degree level or above), monthly income (<£2000 vs ≥£2000), Wardle scale (<3 vs = 3), IMD (<5 more deprived vs ≥6).

**Table 1:** Participant demographics (*N* = 1106).

Characteristic	*n* (%)
**Age, mean (SD)**	61.7 (5.1)
**Age range**	54-74
**Gender**	
** Female**	595 (53.8)
** Male**	510 (46.1)
** Prefer to self-describe**	1 (0.1)
**Ethnicity**	
** White**	1065 (96.3)
** Non-White**	41 (3.7)
**Living situation**	
** Living with others**	883 (79.8)
** Living alone**	223 (20.2)
**Education ONS**	
** No qualification**	32 (2.9)
** Up to 4 GCSEs or equivalent**	171 (15.5)
** 5+ GCSEs, 1 A level or equivalent**	207 (18.7)
** Apprenticeship, 2+ A levels or equivalent**	243 (22.0)
** Degree level or above**	453 (41.0)
**Employment status**	
** Employed full-time**	306 (27.7)
** Employed part-time**	180 (16.3)
** Self-employed**	121 (10.9)
** Unemployed**	32 (2.9)
** Looking after home/family**	43 (3.9)
** Unable to work due to long-term illness/disability**	68 (6.1)
** Retired**	356 (32.2)
**Occupation (primary income earner)**	
** Unemployed**	49 (4.4)
** State benefit recipient/casual work**	72 (6.5)
** Semi-/unskilled manual**	89 (8.0)
** Skilled manual**	121 (10.9)
** Supervisory/clerical/junior managerial**	317 (28.7)
** Intermediate managerial/professional**	333 (30.1)
** Higher managerial/professional**	125 (11.3)
**Monthly income (after tax)**	
** <£2000**	471 (42.6)
** ≥£2000**	635 (57.4)
**Financial wealth**	
** <£25 000**	579 (52.4)
** ≥£25 000**	527 (47.6)
**Wardle**	
** <3**	398 (36.0)
** =3**	708 (64.0)
**IMD**	
** <5 (more deprived)**	538 (49.3)
** ≥6 (less deprived)**	554 (50.7)
**Smoking status**	
** Recent (≤1 month)**	266 (24.1)
** Past smoker short-term (1 month—10 years)**	235 (21.3)
** Past smoker long-term (>10 years)**	602 (54.6)

### Regression of lung cancer screening intention from Socio-Structural, smoking status, cognitive and affective variables

Table 2 summarizes the hierarchical logistic regression model of lung cancer screening intention with socio-structural variables (Step 1), smoking status (Step 2), and cognitive and affective psychological variables (Step 3) as predictors. In the first step, socio-structural variables did not significantly predict intention to attend lung screening, χ^2^(7) = 5.41, *P* = .61, with Nagelkerke R^2^ = .01. None of the socio-structural factors, including age, gender, living situation, income, education, deprivation (IMD), or the Wardle scale, were significant predictors of screening intention. The addition of smoking status in the second step did not significantly improve model fit, χ^2^(2) = 3.96, *P* = .14, with Nagelkerke R^2^ = .02. Although individuals who had quit smoking more than 10 years ago showed borderline higher intention compared to recent smokers, this effect was non-significant (OR = 1.69, *P* = .051). The inclusion of cognitive and affective psychological variables in the final step significantly improved model fit, χ^2^(20) = 397.01, *P* < .001, with Nagelkerke R^2^ = .73, Significant positive predictors were attitude (OR = 2.16, *P* = .006), moral norm (OR = 3.89, *P* < .001), descriptive norm (OR = 1.62, *P* = .045), perceived behavioral control (OR = 3.42, *P* < .001), and anticipated regret “if I do not take part” (OR = 2.01, *P* = .021). Significant negative predictors were anticipated regret “if I take part and lung cancer is found and treated” (OR = 0.50, *P* = .029) and fear (OR = 0.64, *P* = .024). Family subjective norms, GP subjective norms, anticipated regret “If I take part and no problems are found,” and fatalism did not significantly predict screening intention (*ps* > .05). Due to high multicollinearity with other norm measures, the general subjective norm variable was excluded from the final model to ensure model stability.

**Table 2:** Logistic regression of screening intention on socio-structural (step 1), smoking status (step 2), and cognitive and affective psychological variables (step 3) *N* = 1088.

Variable	Socio-structural OR (95% CI)	Smoking status OR (95% CI)	Cognitive + affective variables OR (95% CI)
**Step 1—Socio-structural**			
**Age**	1.00 [0.96-1.05]	0.99 [0.95-1.04]	0.95 [0.88-1.03]
**Gender (male)**	1.19 [0.75-1.89]	1.15 [0.72-1.83]	1.38 [0.63-3.02]
**Living situation (living alone)**	0.86 [0.49-1.51]	0.89 [0.51-1.57]	1.68 [0.62-4.52]
**Income monthly (≥£2000)**	1.36 [0.83-2.22]	1.34 [0.82-2.20]	0.73 [0.31-1.73]
**Wardle (=3)**	1.05 [0.65-1.69]	0.99 [0.61-1.61]	1.09 [0.47-2.49]
**IMD (≥6)**	1.10 [0.70-1.75]	1.09 [0.68-1.73]	1.91 [0.87-4.19]
**Education (degree)**	1.22 [0.75-1.97]	1.24 [0.76-2.01]	1.14 [0.49-2.64]
**Step 2—Smoking status**			
**Past smokers short-term (1)**		1.60 [0.84-3.04]	0.75 [0.24-2.31]
**Past smokers long-term (2)**		1.69 [1.00-2.85]	0.91 [0.34-2.46]
**Step 3—Cognitive + affective**			
**Attitude**			2.16** [1.24-3.76]
**Subjective norm family**			1.76 [0.92-3.38]
**Subjective norm GP**			1.01 [0.58-1.73]
**Moral norm**			3.89** [2.30-6.59]
**Descriptive norm**			1.62* [1.01-2.59]
**Perceived behavioral control**			3.42** [1.98-5.89]
**Anticipated regret normal results**			1.89 [0.82-4.35]
**Anticipated regret no attendance**			2.01* [1.11-3.62]
**Anticipated regret abnormal results**			0.50* [0.27-0.93]
**Fear**			0.64* [0.44-0.94]
**Fatalism**			0.73 [0.41-1.30]
**Model χ²**	5.406	9.363	397.012**
**−2 Log likelihood**	586.230	582.273	194.624
**df**	7	9	20
**Nagelkerke *R*²**	.012	.020	.729

Gender: 0 females, 1 males; Living situation: 0 living with others, 1 living alone; Income monthly: 0 < £2000, 1 ≥ £2000; Wardle: 0 < 3, 1 = 3; Education: 0 without degree, 1 with degree; Smoking status: 0 recent smoker, 1 past smoker (short-term), 2 past smoker (long-term). Abbreviations: CI = confidence interval; OR = odds ratio.

* *P* < .05.

** *P* < .01.

### Thought listings

Our secondary aim was to examine the thoughts generated in response to the lung cancer screening invitation and identify the specific thoughts that underlie the significant cognitive and affective predictors of intention. Overall, fifteen thought types were identified. The most frequent positive thoughts related to the benefits of early detection of cancer and improved treatment outcomes (34.4%), followed by receiving peace of mind and clarity (26.4%). The most common negative thoughts concerned worry about diagnosis, receiving bad news, or waiting for results (21.7%), followed by doubts about program implementation and efficacy (4.8%). Participants also generated counter-arguments, such as discounting personal risk (eg, “I thought after 5 years we were supposed to have the same risk as a non-smoker”) or diminishing the psychological costs (eg, “Not knowing is worse than worry”). Table 3 presents example items and definitions for each type of thought, along with their observed frequencies.

**Table 3:** The Fifteen coded thought subtypes and their frequencies *N* = 1106.

Thought subtype	Definition	Example thought	Total sample (%)
**Counter-positive**	Counter-arguments of screening benefits and minimization of personal cancer risk	“*I thought after 5 years we were supposed to be same risk as non-smoker*.”	4.8
**Practical barriers**	Concerns about screening logistics, including travel, location, scheduling	“*It’s hard for me to travel to hospital appointments.*”	0.6
**Personal concerns**	Fear or uncertainty about the consequences of screening, including radiation exposure, invasive follow-up treatments, and mental health impact	“*I’m worried about radiation exposure.*”	2.6
**NHS concerns**	Skepticism about NHS funding, resource allocation, capacity, or wait screening times	“*There are already too many delays—I wouldn’t bother.*”	2.4
**Doubts efficacy**	Doubts about program reliability, staff competence, and screening accuracy	“*I wonder whether the initial screening is sufficient.*”	4.8
**Stigma smoking**	Feelings of stigma/embarrassment/shame linked to smoking history	“*I would feel a bit of embarrassment that I smoke.*”	1.4
**Worry cancer**	Distress, fear, or worry about being diagnosed with cancer, receiving bad news, or anxiety during the wait for results	“*I’m scared of what they might find.*”	21.7
**Early detection**	Belief that screening enables early detection and improves treatment outcomes	“*This will make early detection and therefore treatment much easier*.”	34.4
**Peace of mind**	Belief that screening provides reassurance, clarity, or peace of mind	“*It would give me peace of mind that I still have healthy lungs.”*	26.4
**Prevention**	Value of the preventative/proactive aspects of the test, including reported motivation to adopt healthier habits like smoking cessation	“*It would prompt me to make more healthy lifestyle choices*.”	12.2
**Saving lives**	Explicit endorsement of screening as a way to save lives	“*It will help to save so many lives by doing this.*”	3.3
**NHS benefits**	Screening seen as good use of NHS resources and valuable public health initiative.	“*It has the potential to save the NHS money in the long run.*”	2.8
**Personal history**	Family or personal cancer history motivating screening attendance	“*I lost a close family member to lung cancer—bring on the screening!*”	1.6
**Positive aspects**	Statements about the free, voluntary, and non-judgmental nature of the test	“*It does not cost anything as it is free.*”	1.7
**Counter-negative**	Counter-arguments of concerns or drawbacks associated with screening, particularly around worry or perceived risk	“*Not knowing is worse than worry.*”	6.4

The table shows the types of thoughts elicited in response to the screening invitation in the total sample and their frequencies.

### Regression of psychological cognitive and affective variables from thought listings

Follow-up multiple linear regressions examined which thought subtypes predicted the cognitive and affective significant predictors of screening intention (see Table 4). All models were significant, with R^2^ values ranging from .03 to .15. For attitude, counter-positive thoughts about dismissing the benefits of screening or minimizing their own cancer risk (*B* = −0.61, *P* < .001), concerns about personal impact (*B* = −0.62, *P* < .001), NHS system concerns (*B* = −0.52, *P* < .001), doubts about program implementation and efficacy (*B* = −0.47, *P* < .001), and worry about cancer (*B* = −0.23, *P* < .001) were associated with lower perceived screening effectiveness, whereas early detection perceived benefits (*B* = 0.14, *P* = .003), peace of mind (*B* = 0.23, *P* < .001), and personal/family history of cancer (*B* = 0.38, *P* = .034) were positively associated with more favorable attitudes toward screening. For moral norm, counter-positive thoughts minimizing cancer risk (*B* = −0.75, *P* < .001), personal concerns (*B* = −0.52, *P* = .012), NHS system concerns (*B* = −0.80, *P* < .001), and doubts about program implementation and efficacy (*B* = −0.62, *P* < .001) were negative predictors, while early detection benefits (*B* = 0.23, *P* < .001), peace of mind benefits (*B* = 0.30, *P* < .001), and personal/family history of cancer (*B* = 0.52, *P* = .038) positively predicted moral obligation to attend screening. Descriptive norms were negatively associated with worry about cancer (*B* = −0.19, *P* = .006), whereas positively associated with perceived peace of mind (*B* = 0.14, *P* = .030). For perceived behavioral control, counter-positive thoughts (*B* = −0.38, *P* < .001), worry about personal impact (*B* = −0.60, *P* < .001), doubts about program implementation and efficacy (*B* = −0.51, *P* < .001), feelings of stigma associated with smoking (*B* = −0.60, *P* = .004), and worry about cancer (*B* = −0.37, *P* < .001) were negative predictors, whereas early detection benefits (*B* = 0.19, *P* < .001) and peace of mind (*B* = 0.21, *P* < .001) positively predicted higher perceived behavioral control to attend screening.

**Table 4:** Multiple linear regressions of thought types on cognitive and affective psychological variables *N* = 1106.

	Attitude *B* (95% CI)	Moral Norm *B* (95% CI)	Descriptive Norm *B* (95% CI)	Pbc *B* (95% CI)	Regret no attendance *B* (95% CI)	Regret abnormal *B* (95% CI)	Fear *B* (95% CI)
**Counter-positive**	−0.61** [−0.82 to −0.40]	−0.75** [−1.05 to −0.45]	−0.21 [−0.47 to 0.05]	−0.38** [−0.60 to −0.16]	−0.72** [−0.96 to −0.48]	−0.07 [−0.30 to 0.16]	−0.56** [−0.89 to −0.24]
**Practical barriers**	0.12 [−0.44 to 0.67]	0.33 [−0.47 to 1.23]	0.23 [−0.48 to 0.93]	−0.31 [−0.90 to 0.28]	0.09 [−0.56 to 0.74]	−0.41 [−1.03 to 0.20]	−0.38 [−1.24 to 0.48]
**Personal concerns**	−0.62** [−0.90 to −0.34]	−0.52* [−0.92 to −0.11]	−0.21 [−0.57 to 0.14]	−0.60** [−0.90 to −0.30]	−0.27 [−0.60 to 0.06]	0.34* [0.03 to 0.65]	0.05 [−0.39 to 0.48]
**NHS concerns**	−0.52** [−0.82 to −0.21]	−0.80** [−1.23 to −0.37]	−0.21 [−0.59 to 0.17]	−0.19 [−0.51 to 0.12]	−0.47** [−0.82 to 1.12]	0.13 [−0.20 to 0.46]	−0.59* [−1.06 to −0.13]
**Doubts efficacy**	−0.47** [−0.68 to −0.25]	−0.62** [−0.93 to −0.31]	−0.20 [−0.47 to 0.08]	−0.51** [−0.74 to −0.29]	−0.47** [−0.72 to −0.22]	0.03 [−0.21 to 0.26]	−0.14 [−0.47 to 0.19]
**Stigma smoking**	−0.11 [−0.49 to 0.28]	0.24 [−0.32 to 0.79]	0.02 [−0.47 to 0.51]	−0.60** [−1.01 to −0.19]	0.57* [0.12 to 1.02]	0.90** [0.47 to 1.33]	0.51 [−.08 to 1.11]
**Worry cancer**	−0.23** [−0.34 to −0.13]	−0.05 [−0.21 to 0.10]	−0.19** [−0.33 to −0.06]	−0.37** [−0.49 to −0.26]	0.10 [−0.03 to 0.22]	0.34** [0.22 to 0.46]	0.62** [0.45 to 0.78]
**Early detection**	0.14** [0.05 to 0.24]	0.23** [0.09 to 0.36]	0.10 [−0.02 to 0.22]	0.19** [0.09 to 0.29]	0.14* [0.03 to 0.25]	−0.08 [−0.18 to 0.02]	−0.10 [−0.24 to 0.05]
**Peace of mind**	0.23** [0.12 to 0.33]	0.30** [0.16 to 0.45]	0.14* [0.01 to 0.27]	0.21** [0.10 to 0.31]	0.20** [0.08 to 0.32]	−0.03 [−0.14 to 0.08]	0.17* [0.01 to 0.32]
**Prevention**	0.05 [−0.08 to 0.19]	0.13 [−0.07 to 0.32]	0.03 [−0.14 to 0.20]	0.09 [−0.05 to 0.23]	0.09 [−0.07 to 0.24]	0.22** [0.07 to 0.37]	0.09 [−0.11 to 0.30]
**Saving lives**	0.12 [−0.13 to 0.37]	0.20 [−0.16 to 0.56]	0.21 [−0.10 to 0.53]	0.06 [−0.21 to 0.32]	0.12 [−0.17 to 0.41]	−0.05 [−0.32 to 0.23]	0.41* [0.02 to 0.79]
**NHS benefits**	0.04 [−0.23 to 0.31]	−0.13 [−0.52 to 0.26]	0.22 [−0.13 to 0.56]	−0.04 [−0.32 to 0.25]	−0.09 [−0.40 to 0.23]	−0.02 [−0.32 to 0.27]	0.12 [−0.30 to 0.54]
**Personal history**	0.38* [0.03 to 0.72]	0.52** [0.03 to 1.02]	−0.00 [−0.44 to 0.44]	0.25 [−0.11 to 0.62]	0.46* [0.06 to 0.86]	0.11 [−0.27 to 0.49]	0.42 [−0.12 to 0.95]
**Positive aspects**	0.11 [−0.23 to 0.45]	−0.10 [−0.58 to 0.39]	0.14 [−0.29 to 0.56]	0.26 [−0.10 to 0.62]	−0.13 [−0.53 to 0.26]	−0.29 [−0.66 to 0.08]	−0.37 [−0.89 to 0.16]
**Counter-negative**	−0.02 [−0.20 to 0.16]	−0.14 [−0.39 to 0.12]	0.06 [−0.17 to 0.29]	−0.06 [−0.25 to 0.13]	−0.12 [−0.09 to 0.33]	0.08 [−0.12 to 0.28]	−0.21 [−0.48 to 0.07]
**Model *F***	12.485**	8.357**	2.343**	12.556**	7.066**	5.360**	6.447**
***df***	15	15	15	15	15	15	15
**Model *R* ^2^**	0.15	0.10	0.03	0.15	0.09	.07	.08

* *P* < .05.

** *P* < .01.

Anticipated regret for not attending the screening was negatively associated with counter-positive thoughts (*B* = −0.72, *P* < .001), NHS system concerns (*B* = −0.47, *P* = .009), and doubts about program efficacy (*B* = −0.47, *P* < .001), while positively associated with perceived early detection benefits (*B* = 0.14, *P* = .013), peace of mind (*B* = 0.20, *P* < .001), personal/family history of lung cancer (*B* = 0.46, *P* = .025), and feelings of stigma (*B* = 0.57, *P* = .013). For anticipated regret for attending screening and receiving abnormal results, concerns about health risks and personal impact (*B* = 0.34, *P* = .034), feelings of stigma associated with smoking (*B* = 0.90, *P* < .001), worry about cancer (*B* = 0.34, *P* < .001), and perceived preventative and proactive health beliefs (*B* = 0.22, *P* =.004) were all significant positive predictors. Finally, fear was negatively associated with counter-positive thoughts (*B* = −0.56, *P* < .001) and NHS system concerns (*B* = −0.59, *P* = .012), whereas worry about cancer (*B* = 0.62, *P* < .001), perceived peace of mind (*B* = 0.17, *P* = .035), and beliefs about the value of screening in saving lives (*B* = 0.41, *P* = .038) positively predicted higher fear of getting cancer.

## Discussion

The present study provides one of the most comprehensive accounts to date of the psychological factors shaping lung cancer screening intention. Using a large UK sample of screening-eligible adults, who had no prior experience of lung screening, we included a comprehensive set of cognitive and affective variables alongside an assessment of spontaneous thoughts in response to a lung screening invitation for the ongoing NHS Lung Cancer Screening Program. This allowed us to identify (1) the key psychological predictors of intention to take part in lung cancer screening and (2) the thought processes shaping these predictors, thereby providing insight into how these variables might be targeted in health communications to promote uptake. Screening intention was shaped by both cognitive (moral norms, perceived behavioral control, attitudes) and affective (anticipated regret for nonattendance, anticipated regret for abnormal results, fear) constructs.

### The role of social norms

Moral norm emerged as the strongest predictor of screening intention, with higher perceived moral obligation associated with higher willingness to be screened. This is particularly noteworthy as moral norms have not previously been examined in the context of lung cancer screening, nor have they, to the best of our knowledge, been examined in any other cancer screening context. Screening encapsulates different moral obligation components (eg, responsibility toward close others, to society, and to the NHS as a custodian of a free program) that might ultimately shape screening behaviors. Indeed, consistent with this, thought analyses revealed that personal/family history of cancer was positively associated with moral norms, suggesting that prior personal or familial experience with cancer partly explains this relationship. This might be because past experiences with cancer might make one reflect on the value of screening if, for example, they or their loved ones have survived cancer, or if someone close to them has passed away and screening might have prevented such an outcome, thereby amplifying feelings of internalized moral responsibility to be screened. This also supports theoretical accounts that moral obligation is heightened when responsibility to oneself or family is salient,^32^^,^^33^ but crucially extends these models to lung cancer screening. On the other hand, moral obligation was undermined by skeptical beliefs about the NHS system and the implementation and efficacy of the screening program, suggesting that when mistrust and doubts are present, then feelings of moral obligation to be screened are subverted, highlighting the need to strengthen trust in the NHS and address program efficacy concerns.^14^^,^^15^ Interestingly, moral norm was also negatively associated with counter-positive thoughts minimizing personal cancer risk and screening benefits, implying that individuals with lower perceived moral obligation to be screened are more likely to engage in maladaptive fear reduction strategies of avoidance and reactance.^45^ Taken together, the findings indicate that moral norm is an important psychological construct to be leveraged in communication intervention design aiming to increase lung screening participation. Interventions might do so by framing screening attendance to emphasize responsibility and shared benefit (eg, to oneself, to family), highlighting screening benefits, while countering skeptical thoughts (eg, by providing clear, transparent information about how screening works and addressing program concerns). Future studies should also aim to further unpack different sources of moral norms and test whether the findings will translate to other screening contexts.

Descriptive norms (perceived behavior of similar others) also emerged as a positive predictor of intention, consistent with past research in other screening contexts,^30^^,^^31^ but to our understanding, this is the first study to demonstrate that descriptive norms exert independent effects on lung cancer screening intention. Thought analyses revealed that descriptive norms were negatively associated with cancer worry and positively with peace of mind benefits, indicating that individuals who spontaneously focus on feared outcomes may believe that others are similarly avoidant or hesitant to attend screening, while those who focus on the screening benefits of peace of mind and reassurance are more likely to believe that similar others will attend the screening. This suggests that descriptive normative interventions should be framed positively, highlighting how similar others attend screening and feel reassured, reinforcing positive social norms.^31^

Subjective norms, relating to family and GP endorsement of taking part in screening, did not emerge as significant predictors of lung cancer screening intention, despite substantial evidence linking subjective norms to both intentions and behaviors in other cancer screening contexts^23^^,^^25^^,^^26^^,^^46^ and despite recent qualitative and quantitative findings in the lung screening context looking at subjective norms in isolation.^12^^,^^13^^,^^20^ Taking everything together, the findings provide novel evidence for the roles of moral and descriptive norms on lung cancer screening intention and suggest that perceived behavior of similar others (descriptive norms) and personal moral obligation (moral norm) are more important than perceived expectations of approval from specific referents in this context, reinforcing arguments for extending TPB’s normative components to move beyond subjective norms.^47^

### The role of perceived behavioral control

Perceived behavioral control emerged as the second strongest predictor of screening intention, suggesting that individuals with higher perceived confidence to take part in lung cancer screening were more likely to intend to be screened. This is consistent with past research showing that PBC is associated with screening intention and behavior in other screening contexts,^23^^,^^25^^,^^26^ but has not previously been examined in the context of lung cancer screening. The finding that PBC is an important determinant of motivation in the lung screening context deserves comment, given the logistical ease of the initial screening (a telephone call with a health professional). Follow-up thought analyses showed that perceived behavioral control was negatively associated with concerns about embarrassment and stigma, worry about cancer, personal concerns (eg, radiation exposure), and doubts about program implementation and efficacy. Findings thus suggest that psychological rather than practical barriers—particularly fear of cancer, stigma, and mistrust—may impede screening participation.^48^ Interventions aiming to increase perceived ability to attend lung screening should therefore focus on increasing efficacy to cope with these negative emotions and reducing psychological barriers by addressing stigma in communication using sensitive, inclusive language,^14^^,^^15^ providing reassurance to address cancer-related worry, while highlighting program reliability to offset efficacy concerns and emphasizing screening benefits.

### The role of affect

Affective constructs also played a critical role in predicting screening intention. Anticipated regret about not taking part in screening positively predicted intention, replicating evidence from other screening contexts that inaction regret predicts cancer screening over and above TPB variables,^35^^,^^36^ but extends these findings to the context of lung cancer screening. Thought content analyses revealed that inaction regret was negatively associated with counter-positive thoughts minimizing personal cancer risk and screening benefits, and doubts about the NHS, program implementation and efficacy, suggesting that skeptical beliefs and tendency to minimize one’s own risk undermine inaction regret. This is aligned with theoretical predictions that individuals may employ a range of maladaptive coping strategies directed at discounting evidence or denying threat in order to minimize unpleasant emotions as a result of facing the threat.^49^ On the other hand, inaction regret was positively associated with personal/family history of cancer, indicating that regret might arise when inaction conflicts with salient personal or familial experiences. Paradoxically, inaction regret was also positively associated with smoking-related stigma, potentially reflecting complex feelings of shame and self-blame^50^ that might be amplified in the case of inaction for an opportunity perceived as personally relevant or corrective.

Conversely, anticipated regret about taking part in screening and receiving abnormal results emerged as a negative predictor of screening intention, consistent with the theoretical prediction from protection motivation theory that high response costs might prompt maladaptive avoidant coping responses^37^ and providing the first evidence for this form of anticipated regret in the lung cancer screening context. Interestingly, smoking-related stigma emerged as the thought most strongly and positively associated with anticipated regret for abnormal results, suggesting that anticipated feelings of blame from others might be further exacerbated in the case of a lung cancer diagnosis where the role of smoking, and therefore of personal responsibility, might be implicated as the main cause, thus fueling maladaptive avoidant responses to avoid facing a coping response that is deemed difficult and psychologically costly.^49^ Taken together, the findings indicate that anticipated regret can function as both a motivator *and* a barrier for lung cancer screening, underscoring the need to consider multiple regret pathways in the screening context.^34^ Communication strategies should therefore primarily focus on leveraging the inaction regret pathway and frame messages around the risk of “missing a valuable opportunity” (loss framing),^51^ while countering skepticism and efficacy concerns and highlighting screening benefits, rather than focusing on anticipated regret for abnormal results, which may further exacerbate fear and avoidant responses.^11^

Finally, fear of cancer also emerged as a significant negative predictor of intention, extending prior research in the uptake context showing that higher fear of cancer may act as a deterrent to lung cancer screening through the mechanism of avoidance^11^^,^^14^ by moving beyond qualitative designs and samples focusing only on non-attenders. Thought analyses revealed that worry about cancer was unsurprisingly the thought most strongly associated with higher fear. Fear was also negatively associated with NHS system concerns and counter-positive thoughts minimizing personal risk and dismissing screening benefits, consistent with maladaptive fear reduction strategies.^45^ Interestingly, fear was also positively associated with the perceived screening benefit of saving lives. This may reflect a nuanced cognitive mechanism whereby emphasizing screening benefits, such as the potential for saving lives, might inadvertently increase salience of cancer risk and amplify already existing concerns by bringing to mind the prospect of mortality. Given the findings and the threat appeal literature recommendations that interventions targeting threat appraisals should be avoided unless paired with elements aimed at increasing response and self-efficacy,^45^ communication interventions should therefore seek to normalize worry, pair it with reassurance, stress efficacy and actionable steps, and avoid excessive threat framing that could trigger avoidance.^11^^,^^48^

### Socio-Structural and smoking status predictors of screening intention

Socio-structural factors such as age, gender, education, income, deprivation, and living arrangements did not predict lung cancer screening intention in the present sample. Long-term past smokers who had ceased smoking more than 10 years ago reported somewhat higher intentions to attend screening compared to those who had smoked cigarettes during the previous month, but this effect did not reach significance in the logistic regression. This aligns with research showing no associations between socio-demographics, smoking status, and lung cancer screening intention,^15^ despite consistent associations with actual uptake behaviors.^9^^,^^11^^,^^52^ Exploratory analyses of our sample revealed differences in the types of thoughts generated by recent and former smokers; recent smokers were more likely than long-term former smokers to endorse the benefits of taking part to help motivate cessation or lifestyle change (16.9% vs. 10.6%), but were less likely than long-term former smokers to endorse the benefits of early detection and treatment (26.7% vs. 38.4%). Findings suggest that current and past smokers may be more persuaded to take part by different types of message content contained in invitation materials and telephone calls from health professionals. Recent smokers were also more likely to express worry about the outcome of taking part and almost all of those who expressed stigma concerns were recent smokers, underscoring the need for interventions to consider the psychological factors disproportionately affecting these groups, rather than treating smoking history merely as a fixed determinant of behavior. Future longitudinal studies should further unpack these pathways and assess their translation to actual screening behaviors.

### Strengths and limitations

A key strength of our study lies in its design: employing a large, screening-eligible sample of older UK smokers and former smokers who had not previously been invited for lung cancer screening. Research in the United Kingdom context is particularly important given that screening and any necessary treatment are offered free at the point of delivery, as well as the nature of the NHS and the targeted lung screening program, all of which may uniquely shape responses to the screening invitation. By including a comprehensive set of cognitive and affective variables, we captured spontaneous thoughts to a realistic lung cancer screening invitation. This allowed us not only to identify the key psychological predictors of screening intention, but also the thought processes shaping these psychological predictors, thereby informing how these constructs might be targeted in health communication strategies tailored to this specific healthcare and cultural context. Nevertheless, some limitations deserve to be acknowledged. The study was cross-sectional and relied on self-reported intention rather than actual screening behavior. Since invitation materials are received by post, findings inform understanding of how invitees might initially process information about the program and form a decision to accept a phone call from a health professional to assess risk. Future experimental research manipulating the identified constructs^53^ and follow-up prospective studies examining subsequent attendance are needed to establish causal evidence^54^ and confirm whether the cognitive and affective findings will translate to the actual uptake context once rollout is in progress. Second, the study did not employ a pre-post design, which limits our ability to determine whether responses reflect pre-existing beliefs or were shaped by the invitation itself. Given that individuals receive screening invitations and form decisions without prior assessment, this approach was intended to approximate real-world conditions and enhance ecological validity, especially in this context where participants had not previously seen the invitation. Third, although the sample was large and broadly representative of older UK smokers, and 50% of participants were from higher deprivation postal codes, recruitment via an online platform requiring digital literacy may limit generalizability.^55^ Future studies should recruit more diverse participants, particularly from lower socio-economic groups, where evidence shows differential screening participation and barriers.^11^

## Conclusion

This study advances theoretical and practical understanding of motivation to take part in a novel lung cancer screening program by identifying spontaneous responses to reading information about the program. Moral and descriptive norms, perceived behavioral control, and inaction regret emerged as positive predictors of intention and might be promoted in health communications. Lower self-efficacy to take part was driven largely by doubts concerning the ability to cope with cancer worry, feelings of embarrassment or stigma that should be addressed via suggestions as to how to manage these emotions in written and verbal communications. The findings highlight clear leverage points for communication interventions: strengthening moral and descriptive norms, enhancing perceived control by reducing psychological barriers, leveraging inaction regret, and addressing fear, stigma, and mistrust to increase screening participation at this critical stage of national implementation.

## Supplementary Material

kaag031_Supplementary_Data

## Open science transparency statements

(1) This study was not formally registered. (2) The analysis plan was not formally pre-registered. (3) De-identified data, analytic code, and all materials used to conduct the study are available on OSF at: https://osf.io/sk4cj/overview?view_only=cf495ed2a2e941bbafc13355ca86c59d.
