# Supplementary material for: Understanding lung cancer screening motivation: cognitive, affective, and thought-based predictors in a UK screening-eligible sample
Source: Ann Behav Med. 2026 Jun 19;60(1):kaag031. doi: 10.1093/abm/kaag031 (PMC13278838; doi:10.1093/abm/kaag031)
Supplement: kaag031_Supplementary_Data [file kaag031_supplementary_data.docx]

**Supplementary Materials**

**S1. Materials**

**Lung Screening Invitation**

**_________________________________________________________________________**

Lung cancer causes more deaths than any other cancer in the UK. Over the next five years, the NHS will offer a free lung health check to men and women who are registered with a GP in England. If you are aged 55 to 74 and are a current or former smoker, you will receive an invitation. This check can help to spot lung cancer early when it is most treatable. This is often before a person has any symptoms. The check includes an initial appointment with a health professional who will ask about your health and medical history. If the lung health check finds problems with your lung health, you will be referred to your GP and may be invited for a scan to take a detailed picture of your lungs.

Some people find that taking part in a lung health check gives them clarity and peace of mind about lung cancer. Some people also find that it prompts them to make healthier lifestyle choices. They also know that if lung cancer is found, any treatment they may need is being done when it is most effective. Some people find that taking part in a lung health check makes them worry about cancer. They may also start to worry about how other people see them and the effects on their close others. Some people find that they have to undergo checks and unpleasant treatment procedures.

**_________________________________________________________________________**

**Thoughts**

**In the space below, please write down the thoughts that came to your mind as you read the passage above.**

________________________________________________________________

________________________________________________________________

________________________________________________________________

________________________________________________________________

________________________________________________________________

**Attitude**

For me, to take part in a lung health check if it were offered to me in the next 12 months would be… (scoring 1-6)

Stem items:

1. Worthless–worthwhile
2. unnecessary–necessary
3. bad–good
4. unimportant–important
5. unpleasant–pleasant
6. harmful–beneficial
7. ineffective–effective
8. intrusive–non-intrusive
9. embarrassing–comfortable
10. annoying–pleasing
11. foolish–wise
12. useless–useful

**Subjective Norms**

1. If I were offered a lung health check in the next 12 months, most people important to me would think I should take part (1 = Strongly disagree/ 6 = Strongly agree)
2. Most people important to me would approve/disapprove of my taking part in the lung health check if it were offered to me in the next 12 months. (1 = Strongly disapprove/ 6 = Strongly approve)
3. Most people important to me would encourage/discourage me about taking part in a lung health check in the next 12 months (1 = Strongly discourage/ 6 = Strongly encourage)
4. Most people important to me would think it is a good idea for me to take part in the lung health check if it were offered to me in the next 12 months (1 = Strongly disagree/ 6 = Strongly agree)

**Subjective Norms Family**

1. My family would think I should take part in the lung health check if it were offered to me in the next 12 months. (1 = Strongly disagree / 6 = Strongly agree)
2. My family would approve/disapprove of my taking part in the lung health check if it were offered to me in the next 12 months. (1 = Strongly disapprove / 6 = Strongly approve)
3. My family would encourage/discourage me from taking part in the lung health check in the next 12 months. (1 = Strongly discourage/ 6 = Strongly encourage)
4. My family would think it is a good idea for me to take part in the lung health check if it were offered to me in the next 12 months. (1 = Strongly disagree / 6 = Strongly agree)

**Subjective Norms GP**

1. My GP would think I should take part in the lung health check if it were offered to me in the next 12 months. (1 = Strongly disagree / 6 = Strongly agree)
2. My GP team would approve/disapprove of my taking part in the lung health check if it were offered to me in the next 12 months. (1 = Strongly disapprove / 6 = Strongly approve)
3. My GP team would encourage/discourage me from taking part in the lung health check in the next 12 months. (1 = Strongly discourage / 6 = Strongly encourage)
4. My GP would think it is a good idea for me to take part in the lung health check if it were offered to me in the next 12 months. (1 = Strongly disagree / 6 = Strongly agree)

**Descriptive Norm**

Thinking about people of your age and gender that you know, how many of them do you think will take part in a lung health check if it is offered to them within the next 12 months?

(1 = none of them / 5 = almost all of them)

**Moral Norm**

Here are some statements that some people might have about participating in a lung health check. Please indicate the extent to which you agree or disagree with each statement.

Scoring (1 = Strongly disagree / 6 = Strongly agree)

1. It would be morally right for me to take part in a lung health check if it is offered to me.
2. I would feel guilty if I receive an invitation for a lung health check and did not go.
3. I have a moral obligation to take part in a lung health check if it is offered to me.
4. I feel I ought to take part in a lung health check if I get offered it.

**Perceived Behavioural Control**

1. If I were offered a lung health check in the next 12 months, I am unsure/sure whether I would be able to take part in it, even if I wanted to. (1 = very unsure / 6 = very sure)
2. If I were offered a lung health check in the next 12 months, for me to take part in it would be... (1 = totally outside of my control / 6 = totally under my control)
3. If I were offered a lung health check in the next 12 months, I am confident/unconfident that I would be able to take part in it if I wanted to. (1 = very unconfident / 6 = very confident)
4. If I were offered a lung health check in the next 12 months, I am certain that I could overcome any challenges that might prevent me from taking part in it. (1 = very uncertain / 6 = very certain)
5. For me, to take part in a lung health check if it were offered to me in the next 12 months would be... – inconvenient – convenient (1-6)
6. For me, to take part in a lung health check if it were offered to me in the next 12 months would be...- difficult - easy (1-6)
7. For me, to take part in a lung health check if it were offered to me in the next 12 months would be... – burdensome - effortless (1-6)

**Anticipated Regret No Attendance**

If I am offered the chance to take part in the lung health check in the next 12 months and I do not take part, I will feel. . .

Scoring (1-6)

Stem items:

1. Not regretful – regretful
2. Relieved – not relieved
3. Not sorry – sorry
4. Not worried – worried
5. Not ashamed – ashamed

**Anticipated Regret Normal Results**

If I am offered the chance to take part in the lung health check in the next 12 months and I take part, and no problems are found I will feel. . .

Scoring (1-6)

Stem items:

1. Not regretful – regretful
2. Relieved – not relieved
3. Not sorry – sorry
4. Not worried – worried
5. Not ashamed – ashamed

**Anticipated Regret Abnormal Results**

If I am offered the chance to take part in the lung health check in the next 12 months and I take part, and lung cancer is found and treated, I will feel. . .

Scoring (1-6)

Stem items:

1. Not regretful – regretful
2. Relieved – not relieved
3. Not sorry – sorry
4. Not worried – worried
5. Not ashamed – ashamed

**Fear**

The thought of getting lung cancer makes me feel...

Scoring (1 – 6)

1. Not at all scared – very scared
2. Not at all anxious – very anxious
3. Not at all frightened – very frightened
4. Not at all ashamed – very ashamed

**Fatalism**

This section has a list of statements about your views on cancer and its outcomes. Please indicate the extent to which you agree or disagree with each statement.

Scoring (1 = Strongly disagree / 6 = Strongly agree)

1. I think if someone is meant to have cancer, it doesn't matter what kinds of food they eat, they will get cancer anyway.
2. I think if someone has cancer, it is already too late to get treated for it.
3. I think someone can eat fatty foods all their life, and if they are not meant to get cancer, they won't get it.
4. I think if someone is meant to get cancer, they will get it no matter what they do.
5. I think if someone gets cancer, it was meant to be.
6. I think if someone gets cancer, their time to die is soon.
7. I think if someone gets cancer, that's the way they were meant to die.
8. I think getting checked for cancer makes people scared that they may really have cancer.
9. I think if someone is meant to have cancer, they will have cancer.
10. I think some people don't want to know if they have cancer because they don't want to know they may be dying from it.
11. I think if someone gets cancer, it doesn't matter whether they find it early or late, they will still die from it.
12. I think if someone has cancer and gets treatment for it, they will probably still die from the cancer.
13. I think if someone was meant to have cancer, it doesn't matter what doctors and nurses tell them to do, they will get cancer anyway.
14. I think if someone is meant to have cancer, it doesn't matter if they eat healthy foods, they will still get cancer.
15. I think cancer will kill you no matter when it is found and how it is treated.
16. I think if someone is meant to have cancer, it doesn't matter if they don't smoke, they will still get cancer.
17. I think someone can smoke all their life, and if they are not meant to get cancer, they won't get it.

**Screening Intention**

1. I intend to take part in a lung health check if it were offered to me in the next 12 months. (1 = Strongly disagree / 6 = Strongly agree)
2. How likely is it that you would take part in a lung health check if it were offered to you in the next 12 months? (1 = Very unlikely / 6 = Very likely)
3. I intend to take part in a lung health check if it were offered to me in the next 12 months. (1 = Definitely do not intend / 6 = Definitely intend)
4. I plan to take part in a lung health check if it were offered to me in the next 12 months. (1 = Definitely do not plan to / 6 = Definitely plan to)
5. I would definitely go for a lung health check if it was offered to me in the next 12 months. (1 = Definitely will not go / 6 = Definitely will go)
6. I am committed to take part in a lung health check if it was offered to me in the next 12 months. (1 = Definitely not committed to / 6 = Definitely committed to)

**S2. Coding Framework**

**Core Principle**

- Only code thoughts that directly influence screening decisions**.**
- Exclude general opinions unless they explicitly relate to the participant’s own attendance/attitudes etc.

**Coding for Valence**

Each distinct thought should be assigned a score of 1, regardless of its length or complexity. This scoring is applied per thought per valence category (Positive and Negative).

- If a participant expresses one positive and one negative thought, each category receives a count of 1.
- If a participant expresses two negative thoughts, the Negative category receives a count of 2.
- If a single sentence contains multiple distinct thoughts, each is scored separately if they reflect different ideas or sentiments.

**1. Positive Thoughts**

**Definition:** Statements that express the participant’s own beliefs, feelings, or intentions which may increase the likelihood of attending screening, or which highlight perceived benefits or positive outcomes of screening.

**Note:** The statement must be personal to the participant. General endorsements of screening that do not clearly indicate personal relevance or intention should not be coded, unless they are explicitly linked to the participant’s own views or behaviour.

**Examples of coded positive thoughts:**

- *“It would give me peace of mind about my lung health.”*
- *“Early detection makes treatment easier.”*
- *“I can see why others may worry, but for me, finding out whether or not I have cancer outweighs this"*

**What not to code as Positive Thoughts:**

- *“I can see why this might be useful for some people.”* → **Irrelevant**, unless the participant goes on to express personal agreement or intent.
- *“Some people might feel reassured by this.”* → **Irrelevant**, as it lacks a personal element unless explicitly tied to the participant’s own feelings or decisions.
- *“How is the NHS going to afford it/ this is going to cost the NHS a lot.”* → **Irrelevant**, as it lacks a personal element unless explicitly tied to the participant’s own feelings or decisions.

**2. Negative Thoughts**

**Definition:** Statements that reflect the participant’s own thoughts, feelings, or experiences which may decrease the likelihood of attending screening or highlight perceived negative outcomes associated with screening.

**Note:** The statement must be personal. General statements or expressions of understanding about others' experiences should not be coded under this category unless they are explicitly linked back to the participant.

**Examples of coded negative thoughts:**

- *"Health checks worry me."*
- *"I'm scared of what they might find."*

**Examples of what not to code as negative thoughts:**

- *"I can understand why other people might worry."* → **Irrelevant**, unless followed by a personal reflection (e.g., *"...but I don’t feel that way"* or *"...and I feel the same"*).
- *"Some people get anxious about going to the doctor."* → **Irrelevant**, as it does not reflect the participant's own experience.

**THOUGHT CATEGORIES**

Alongside the primary valence coding, secondary codes are applied to capture the content or nature of each thought and to allow for content analysis.

**Coding rule:** Each thought type is coded as 0 if it is absent and 1 if it is present.

**Note:** Thoughts that capture endorsement and intention (either positive or negative) should not be coded as distinct thought types as they are more general.

**Types of Negative Thoughts**

***1. Counter-Positives (0/1)***

**Definition:** Statements that dismiss or downplay the benefits or value of screening or minimise personal cancer risk.

**Example:**

- *“I thought after 5 years we were supposed to be same risk as a non-smoker”*
- *“I am not at risk”*

***2. Worry about Cancer/Diagnosis (0/1)***

**Definition:** Statements that reflect personal emotional distress, fear, or worry about being diagnosed with cancer, receiving bad news, or experiencing anxiety during the wait for results. These feelings are directly tied to perceived psychological risks of attending screening and may reduce likelihood of participation.

**Example:**

- *“I worry about what the test might show”*

***3. Practical/Access Barriers (0/1)***

**Definition:** Statements that describe practical obstacles to participation, including travel, location, scheduling, or administrative processes.

**Example:**

- *“Hospital bureaucracy deters me.”*

***4. NHS System Concerns (0/1)***

**Definition:** Criticism or scepticism regarding NHS funding, resource allocation, capacity, or wait times, only when this is tied to the participant’s screening decision.

**Example:**

- *“Won’t go due to NHS delays.”*

***5. Personal Health Concerns (0/1)***

**Definition**: Fear or uncertainty about the health risks or unintended consequences of screening (e.g. radiation exposure, invasive follow-up treatments, mental health impact).

**Example:**

- *“Worry about scan safety.”*

***6. Doubts about Programme Efficacy (0/1)***

**Definition:** Doubt or mistrust regarding how the screening programme will be delivered, particularly with reference to staff competence or programme reliability.

**Examples:**

- *“Will it be done by trained people?”*
- *“I wonder whether the initial screening is sufficient.”*

**What Not to Code:**

- General NHS or programme criticism without personal relevance (e.g., *“The NHS is underfunded”*).
- Hypothetical or speculative concerns not clearly linked to the participant’s own intentions.
- Questions or statements without an implied effect on behaviour.

***7. Smoking Stigma (0/1)***

**Definition:** Statements reflecting feelings of stigma/embarrassment/shame linked to smoking behaviour that could affect attendance

**Example:**

- *“As a current smoker, I suppose I would feel a bit of embarrassment that I smoke”*

**Types of Positive Thoughts**

**1. Early Detection Benefits (0/1)**

**Definition:** Statements reflecting the perceived early detection value of screening, such early detection of lung cancer or other issues, which increases the chances of successful treatment and better health outcomes.

**Example:**

- *"Early diagnosis...increased chances of successful treatment..."*

**2. Peace of Mind/Reassurance (0/1)**

**Definition:** Statements reflecting that screening provides reassurance, clarity, or peace of mind, either by confirming good health or by addressing worries about possible illness.

**Example:**

- *"It would give me peace of mind that I still have healthy lungs...".*

**3. Prevention/Proactive Health (0/1)**

**Definition:** Statements highlighting screening as a proactive or preventative health measure, emphasising the value of acting before symptoms appear, as well as the value of the test in motivating them to adopt healthier habits like smoking cessation.

**Example:**

- *"* *It would prompt me to make more healthy lifestyle choices.”*

**4. Saving Lives (0/1)**

**Definition:** Statements mentioning the value of screening in saving lives, such as by catching disease early and preventing unnecessary deaths.

**Example:**

- *"This is an amazing way to test for the early symptoms, and it will help to save so many lives by doing this...".*

**5. NHS Benefits (0/1)**

**Definition:** Positive comments about the screening program being a good use of NHS resources, potentially saving money, or being a valuable public health initiative.

**Example:**

- *"It sounds like a very good use of NHS resources. I'd fully support it.".*

**6. Personal/Family Cancer History (0/1)**

**Definition:** Positive attitudes influenced by personal or family experiences with lung cancer or other illnesses, leading to greater appreciation of screening.

**Note:** Code as positive only if linked to intention/value of screening.

**Example:**

- *"I lost a close family member to lung cancer last year - bring on the screening!".*
- *“My dad died from cancer. Had this been around then, he may still be here with us.”*

**7. Positive Aspects of the Test (0/1)**

**Definition:** Statements about the value of the test not captured by other thought types, such as the test is voluntary, free, and that the program is offered without judgment, making it more appealing.

**Example:**

- *“It does not cost anything as it is free.”*

**8. Counter-negatives (0/1)**

**Definition:** Statements that directly refute concerns or drawbacks commonly associated with screening, particularly around worry or perceived risk.

**Example:**

*"Not knowing is worse than worry.”*
